# Bioprospecting the Curculigoside-Cinnamic Acid-Rich Fraction from *Molineria latifolia* Rhizome as a Potential Antioxidant Therapeutic Agent

**DOI:** 10.3390/molecules21060682

**Published:** 2016-06-17

**Authors:** Der Jiun Ooi, Kim Wei Chan, Nadarajan Sarega, Noorjahan Banu Alitheen, Hairuszah Ithnin, Maznah Ismail

**Affiliations:** 1 Nutri-Cosmeceuticals, Nutrigenomics & Nanodelivery Programme, Laboratory of Molecular Biomedicine, Institute of Bioscience, Universiti Putra Malaysia, 43400 Serdang, Malaysia; ooiderjiun@gmail.com (D.J.O.); chankim@upm.edu.my (K.W.C.); sarega5166@gmail.com (N.S.); 2 Department of Cell and Molecular Biology, Faculty of Biotechnology and Biomolecular Sciences, Universiti Putra Malaysia, 43400 Serdang, Malaysia; noorjahan@upm.edu.my; 3 Department of Pathology, Faculty of Medicine and Health Sciences, Universiti Putra Malaysia, 43400 Serdang, Malaysia; hairuszah@upm.edu.my; 4 Department of Nutrition and Dietetics, Faculty of Medicine and Health Sciences, Universiti Putra Malaysia, 43400 Serdang, Malaysia

**Keywords:** *Molineria latifolia* rhizome, ethyl acetate fraction, curculigoside-cinnamic acid-rich fraction, antioxidant activity, oxidative stress, 3T3-L1 preadipocytes

## Abstract

Increasing evidence from both experimental and clinical studies depicts the involvement of oxidative stress in the pathogenesis of various diseases. Specifically, disruption of homeostatic redox balance in accumulated body fat mass leads to obesity-associated metabolic syndrome. Strategies for the restoration of redox balance, potentially by exploring potent plant bioactives, have thus become the focus of therapeutic intervention. The present study aimed to bioprospect the potential use of the curculigoside-cinnamic acid-rich fraction from *Molineria latifolia* rhizome as an antioxidant therapeutic agent. The ethyl acetate fraction (EAF) isolated from *M. latifolia* rhizome methanolic extract (RME) contained the highest amount of phenolic compounds, particularly curculigoside and cinnamic acid. EAF demonstrated glycation inhibitory activities in both glucose- and fructose-mediated glycation models. In addition, *in vitro* chemical-based and cellular-based antioxidant assays showed that EAF exhibited high antioxidant activities and a protective effect against oxidative damage in 3T3-L1 preadipocytes. Although the efficacies of individual phenolics differed depending on the structure and concentration, a correlational study revealed strong correlations between total phenolic contents and antioxidant capacities. The results concluded that enriched phenolic contents in EAF (curculigoside-cinnamic acid-rich fraction) contributed to the overall better reactivity. Our data suggest that this bioactive-rich fraction warrants therapeutic potential against oxidative stress-related disorders.

## 1. Introduction

Redox homeostasis, being a metabolic equilibrium between reduction and oxidation, is important in maintaining normal metabolism by ensuring proper response from the cells to either endogenous or exogenous stimuli. Energy harvesting through cellular redox process releases by-products as reactive species: oxygen (ROS) and nitrogen (RNS). These reactive species are crucial for cell signaling. Overwhelming levels and dysregulation of the reactive species, however, disrupt the delicate balance [1]. The shift towards the oxidized state leads to oxidative stress that has been shown to be implicated in the pathophysiology of several human diseases, including diabetes, cancer, cardiovascular diseases and neurodegenerative diseases [2,3].

As a metabolic organ, adipose tissue participates actively in maintaining energy balance. Nutritional overload triggers redox changes and leads to the expansion of adipose tissue for additional fat stores. Excessive fat storage is associated with the development of metabolic syndrome. The mass expansion of adipose tissue is regulated by the differentiation of preadipocytes into mature adipocytes. The presence of intracellular and extracellular ROS has been shown to promote adipogenic differentiation. Contrarily, restoration of the homeostatic redox balance by the use of antioxidant impedes the process of adipogenesis [4,5]. Strategies to restore redox balance, particularly the exploration of potent compounds from plants, have thus become the focus of therapeutic intervention [6].

Plant secondary metabolites, including phenolic compounds (tannins, phenolic acids and flavonoids), nitrogen compounds (alkaloids and amines), terpenoids and carotenoids, are originally produced by the plant in response to specific environmental stimuli. Many of the secondary metabolite constituents have been proven beneficial to improve health status [7]. Much research attention, however, has focused on the use of polyphenols for promoting human health and disease prevention. Plant polyphenols have been demonstrated to be potent antioxidants and offer beneficial effects against the development and progression of various pathological conditions. The synergistic effect of polyphenols has been purported to contribute towards their better efficacy, where efforts to isolate individual bioactive components may be rendered irrelevant due to the plethoric amount present [8]. The preparation of a standardized bioactive-rich fraction with consistent quality and effect has thus been suggested to maximize the potential of phytochemicals [9].

*Molineria latifolia* (Dryand. ex W.T.Aiton) Herb. ex Kurz or its synonyms, *Aurota latifolia* (Dryand. ex W.T.Aiton) Raf. and *Curculigo latifolia* Dryand. ex W.T.Aiton, is a rhizome geophyte categorized under genus *Molineria* and family *Hypoxidaceae* [10]. Propagating by underground tubers, *M. latifolia* can be found distributed from China (Guangdong) to Malaysia. Locally known as lemba in Malaysia, its rhizome is traditionally used to cure jaundice and to aid in wound healing. Scientific findings had also revealed the hepatitis B virus inhibitory effect of the plant [11] and the anti-diabetic properties of the plant crude extract in our laboratory [12].

The present study aimed to prepare a novel high antioxidative fraction from *M. latifolia* rhizome and to evaluate its therapeutic potential to restore redox balance in a preadipocyte cell model. The phenolic contents of rhizome extract and its fractions were determined spectrophotometrically and by high-performance liquid chromatography with diode-array detection (HPLC-DAD). The antioxidant properties were assessed by employing multiple chemical-based assays with varying fundamental principles. Besides, the protein-phenolic interactions were examined by evaluating the non-enzymatic protein glycation. The fraction-based cell viability assessment and effects on cellular antioxidant defense systems were further tested on 3T3-L1 preadipocytes, a widely-used cellular model for the study of adipocyte development and metabolism. Correlational analysis was performed to determine the relative contribution of identified phenolic compounds to the antioxidant activities.

## 2. Results and Discussion

### 2.1. Extract/Fraction Yield, Total Phenolic Content and Total Flavonoid Content

The yield, total phenolic content (TPC) and total flavonoid content (TFC) of RME and solvent-partitioned fractions of *M. latifolia* rhizome are shown in Table 1. The yield of rhizome methanolic extract (RME) was 7.95 g/100 g in relation to the dry weight of the raw material. Further bio-guided fractionation of RME yielded fractions with different yields, ranging from 0.71 to 42.54 g/100 g in relation to the dry weight of the residue. Descending order in the yield values was observed in the sequence of aqueous fraction (AQF) > *n*-butanol fraction (BUF) > ethyl acetate fraction (EAF) > hexane fraction (HXF) (*p* < 0.05). The differences in the extraction yield, however, did not correlate with TPC and TFC. Although having the second lowest fraction yield, EAF observed the highest phenolics and flavonoid content. The TPC of EAF was approximately 3.9-, 624.3-, 7.5- and 40.5-fold (*p* < 0.05) higher than that of RME, HXF, BUF and AQF, respectively. Additionally, TFC for EAF was respectively 3.5-, 426.5-, 7.1- and 47.4-fold (*p* < 0.05) higher when compared to the aforementioned. On the contrary, AQF, which had the highest fraction yield, recorded low TPC and TFC.

Compound extractability is largely subjected to the differences in the solubility and polarity of the extracted compounds, as well as the solvents. Significantly lower yield in the non-polar HXF indicated low oil content, while a higher yield in the polar AQF proposed concomitant extraction of macronutrients, including protein and carbohydrates, into the fraction [13]. The high concentration of phenolic and flavonoid extracted by the EAF suggested that most of the compounds were of intermediate polarity. Flavonoids and related phenolic compounds have been shown to exert a multiplicity of biological functions against various human pathological conditions through their potential effect on cellular viability, interactions with proteins and antioxidant activity [14]. Thus, the antioxidant actions, cell viability assessments and protein-phenolic interactions of the extract and fractions were further evaluated in the present study.

### 2.2. Phenolic Compositions by HPLC-DAD Analysis

**Figure 1:**
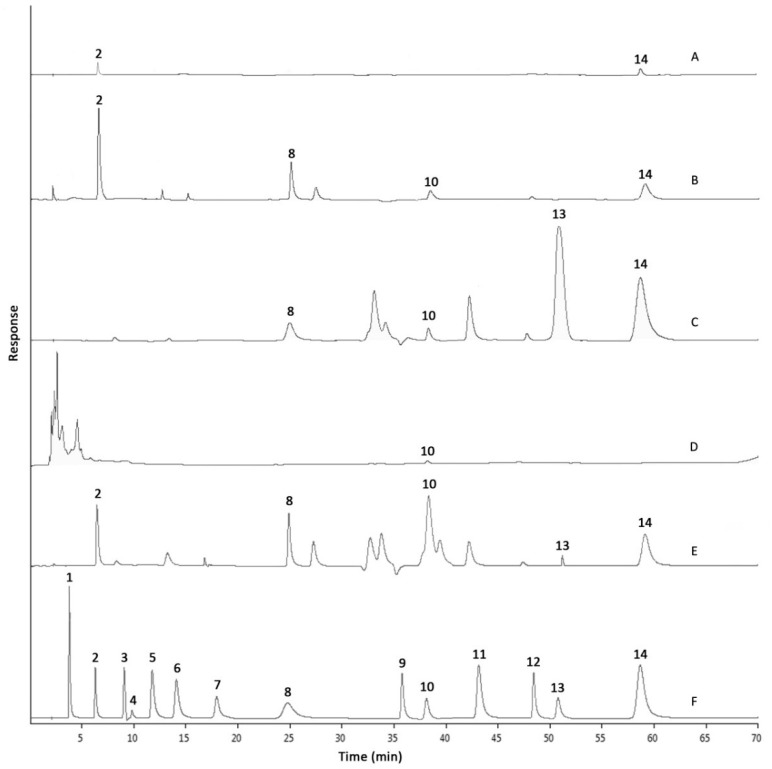
Representative HPLC chromatograms. A: AQF; B: BUF; C: EAF; D: HXF; E: RME; F: mixed phenolic standard. Peaks: 1, gallic acid; 2, protocatechuic acid; 3, *p*-hydroxybenzoic acid; 4, gentisic acid; 5, chlorogenic acid; 6, vanillic acid; 7, caffeic acid; 8, syringic acid; 9, *p*-coumaric acid; 10, ferulic acid; 11, sinapic acid; 12, salicylic acid; 13, curculigoside; 14, cinnamic acid.

**Figure 2:**
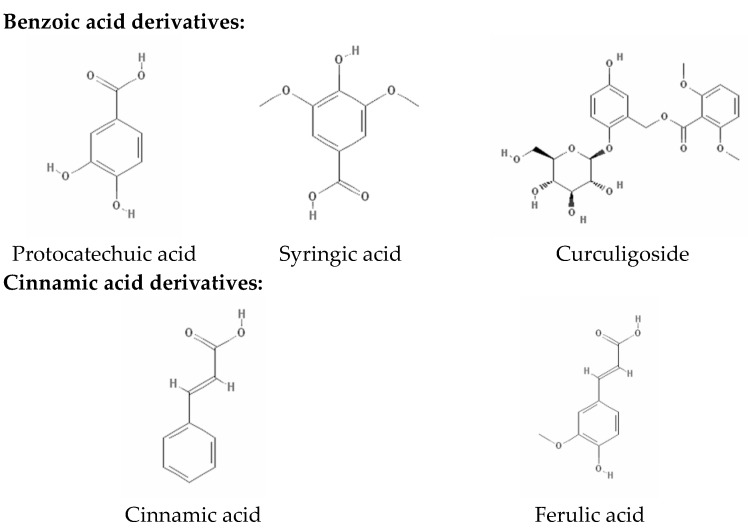
Chemical structures of the identified phenolic compounds [19].

The representative chromatograms of the phenolic standards (3.91 μg/mL each) and sample (500.00 μg/mL each) separations are shown in Figure 1. In general, five major phenolic compounds, in the descending sequence of cinnamic acid > curculigoside > syringic acid > ferulic acid ≥ protocatechuic acid (*p* < 0.05) were identified in RME (structures shown in Figure 2). The concentrations of various phenolic compounds in RME and its derived fractions as determined by HPLC-DAD analysis are revealed in Table 2. Fractionation by solvents of varying polarity imposed differing effects on the presence of the compounds. In particular, the dissolvability of compounds depends on the polarity, polarizability, dipole moment and hydrogen-bonding capacity of a solvent. Both HXF and AQF recorded low amounts of phenolic contents. The observations might partly be attributable to the fact that phenolics are not soluble in the non-polar hexane. At the same time, most of the phenolics might have been extracted through fractionation with the polar aprotic ethyl acetate and polar protic *n*-butanol.

Concentrations of curculigoside and cinnamic acid in EAF were respectively increased up to 14.0- and 1.8-fold (*p* < 0.05) in comparison to that of RME. Traces of ferulic acid and syringic acid were also identified in EAF. BUF observed a 2.6-fold (*p* < 0.05) higher concentration of protocatechuic acid when compared to RME. However, the presence of other compounds in BUF, including ferulic acid, cinnamic acid and syringic acid, were lower. The sum of phenolic contents detected by HPLC corresponded to TPC determined spectrophotometrically, in which EAF > RME ≥ BUF > AQF > HXF (*p* < 0.05); albeit lower total phenolics from HPLC was recorded. The non-specificity of the Folin–Ciocalteu assay and restrictions of phenolics standards used in the HPLC analysis might have contributed to the variations observed [15].

The total phenolic content found in EAF was approximately 3.9-fold (*p* < 0.05) higher than the crude extract where it was being derived. This observation implied that EAF had a high selectivity for the major phenolic constituents present in RME, in agreement with the findings of Mariod *et al.* [16] and Lee *et al.* [17], of which both reported more prominent levels of total phenolics by ethyl acetate fractionation. The logarithm of the partition constant (log *P*) provides the estimation of the molecular hydrophobicity and hydrophilicity. Curculigoside and cinnamic acid belong to an intermediate polarity range, with respective log *P* -values of −0.024 and 2.14. Hence, this explicated the enrichment of both molecules in the ethyl acetate solvent [18].

### 2.3. Antioxidant Activities

The antioxidant activities of *M. latifolia* RME and its fractions, as shown in Table 3, were assessed by various antioxidant assays based on different mechanism of action. The radical-eradicating capability of RME and its derived fractions was evaluated by both the 1,1-diphenyl-2-picrylhydrazyl (DPPH) radical and 2,2′-azino-bis(3-ethylbenzthiazoline-6-sulphonic acid) (ABTS) radical cation scavenging assays. The results from both assays demonstrated the same trend in the order of trolox ≥ EAF ≥ BUF ≥ RME > HXF > AQF (*p* < 0.05). EAF was found to have the highest effectiveness against the two stable radicals, with a trolox-comparable capability in scavenging the DPPH radical. Contrarily, both AQF and HXF showed the lowest antiradical activity, with respective IC_50_ values of 707.41 ± 143.29 and 495.47 ± 24.04 μg/mL against the DPPH radical and 2537.98 ± 379.22 and 1588.50 ± 261.61 μg/mL against the ABTS radical cation. The results, in accordance with Das *et al.* [20], demonstrated that phenolic compounds’ enrichment greatly improved the capability of the extract or fraction in scavenging the stable radicals.

The beta-carotene-linoleate model system was employed to measure the effectiveness of the extract and fractions against heat-induced lipid peroxidation. Initialization of lipid peroxidation by reactive oxygen species triggers a self-propagating chain reaction mechanism that leads to the destruction of membrane lipids and tissue damage. Surprisingly, the present results demonstrated that RME (3.97 ± 0.22 μg trolox equivalent (TRE)/mg extract) outperformed all of the derived fractions in the beta-carotene bleaching (BCB) assay. The results among the fractions varied from 0.57 ± 0.04–2.34 ± 0.06 μg TRE/mg fraction, with a decreasing order of EAF > BUF > AQF > HXF (*p* < 0.05). In comparison, the data suggested that RME and EAF might be efficient in preventing the damage of biomolecules, including polyunsaturated fatty acids, lipoprotein and DNA.

The polar paradox theory states that hydrophilic antioxidants are more active in a pure oil system, while lipophilic antioxidants are more effective in an oil-in-water emulsion system. Nevertheless, the concept contradicted the present findings, as the constituents of EAF and RME ranged from intermediate to high polarity. The nonlinear hypothesis, on the other hand, suggests that the alkyl esters’ chain length of phenolics could affect the antioxidant capacity in emulsion. In the present case, the presence of naturally-occurring polymeric phenolics of midmolecular weight in RME and EAF might impart their inhibitory effects against lipid peroxidation. More experimental data, however, are needed to further substantiate the findings [21].

Reducing properties are generally associated with the potential of compounds in donating a hydrogen atom, thus breaking down the free radical reaction chain. The ferric reducing antioxidant power (FRAP) assay demonstrated the reducing potential of the extract and fractions reacting with Fe^3+^. In accordance with the antiradical assays, EAF was found to possess better reducing power with a FRAP value of 308.94 μg TRE/mg fraction, approximately 1.5-, 2.4-, 1.6- and 5.4-fold (*p* < 0.05) higher, in comparison with RME, HXF, BUF and AQF.

The availability of transition metal ion Fe^2+^ in a biological system catalyses the production of a variety of potent oxidizing species, thereby initiating lipid peroxidation. Simultaneous presence of Fe^2+^, Fe^3+^, ferryl-oxo and perferryl-oxo intermediates perpetuates the rate and extent of iron-induced pro-oxidative transition [22]. Chelation of metal ions, measured by the iron chelating activity assay, could therefore reduce the formation of reactive oxygen species. The results from the assay ranged from 1.90 ± 0.04–6.13 ± 1.35 mg/mL and reduced in the following manner: EAF ≥ AQF ≥ BUF > HXF ≥ RME (*p* < 0.05). Both FRAP and iron chelating activity assays revealed the role of EAF acting as an effective secondary or preventive antioxidant in iron-initiated lipid peroxidation.

Other than iron chelating activity and the BCB assay, correlational statistics revealed distinct correlation (0.775–0.974; *p* < 0.05) among the phenolic content and antioxidant activities of RME and its derived fractions. Being classified as primary antioxidants, most phenolic compounds act as free radical scavengers that delay or inhibit the propagation of redox signaling cascades [23]. The weak correlation between phenolic content and iron chelating activity suggested the reaction of bioactive compounds other than phenolics, in accordance with the report by Chan *et al.* [24]. The amount of phenolic content, however, bestows primary antioxidant activity and a protective property against oxidative stress. Report by Hatia *et al.* [25] stated the antioxidant and anti-inflammatory effects of polyphenols on preadipocytes under hydrogen peroxide (H_2_O_2_)-induced oxidative stress. Such observations have been reported in conditions involving pre-treatment and co-exposition of both polyphenols and pro-oxidants.

Thus, enrichment of phenolic content in EAF (particularly curculigoside and cinnamic acid) by simple ethyl-acetate fractionation could potentially heighten the antioxidant and anti-oxidative effect for the reinstatement of redox balance. Evaluation of protein-phenolic interactions and cellular antioxidant defenses was performed to further validate the hypothesis on biological functions.

### 2.4. Non-Enzymatic Anti-Glycation Activities

Non-enzymatic modifications of proteins are accompanied by the generation of free radicals via glucose auto-oxidation. The modification may lead to functional damage of the protein and pathogenic consequences. In the present study, anti-glycation activity was evaluated by the ability of the extract or fractions to inhibit the formation of fluorescent advanced glycation end products in both glucose- and fructose-mediated protein glycation model systems. The glycating ability of monosaccharide varies among different concentrations and types of simple sugars. Fructose has been demonstrated to possess a faster glycation rate and protein cross-linking effects when compared to glucose [26].

**Figure 3:**
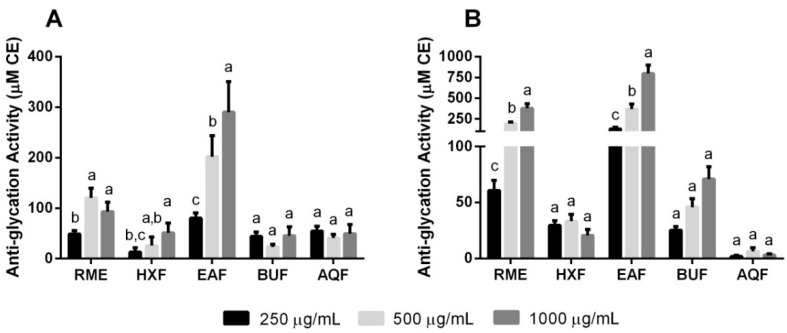
Anti-glycation activities in (**A**) BSA/glucose and (**B**) BSA/fructose model systems. Data are expressed as the mean ± the standard deviation (*n* = 6). Different letters indicate significant differences (*p* < 0.05) within the different treatment concentrations. CE, catechin equivalent.

The results from the present study (Figure 3) demonstrated that EAF exhibited significantly higher glycation inhibitory activities in a dose-dependent manner on both tested model systems. On the other hand, RME only showed its dose-dependent anti-glycation efficacy in the bovine serum albumin (BSA)/fructose model. A correlational study revealed significant correlation (0.880–0.975; *p* < 0.05) between phenolic/flavonoid contents and anti-glycation activities in both glycation models. The capacity of phenolic acids to modulate early stages of protein glycation has been of particular interest. Report by Vlassopoulos *et al.* [27] presented the inhibitory role of phenolic pre-treated protein in a physiologically-relevant glycation system.

Cinnamic acid, being one of the major phenolics found in EAF, along with its derivatives has previously been shown to exhibit effective anti-glycation activity, potentially due to the hydroxy and methoxy substituent groups found in the structure [28]. Furthermore, the review by Ozdal *et al.* [29] stated that strong BSA-binding affinity by cinnamic acids leads to significant diminution in the BSA α-helix structure. The structural changes to the protein may subsequently afford protective effects against protein oxidation. Briefly, these findings suggested that EAF exhibited glycation inhibitory activity through protein-phenolic interactions. However, the exact mechanism of how the polyphenols react with the protein is subjected to further investigation.

### 2.5. Cell Viability Assessment

The effect of RME and its derived fractions on cellular metabolic functions were investigated using both (3-(4,5-dimethylthiazol-2-yl)-2,5-diphenyltetrazolium bromide) (MTT) and neutral red uptake (NRU) assays. The MTT assay measures the conversion of soluble tetrazolium salt to an insoluble formazan precipitate in active mitochondria of viable cells. The NRU assay evaluates the lysosomal uptake of neutral red dye by viable cells and their membrane integrity. The concentrations of each extract or fractions (μg/mL) required to produce the respective 20% or 50% reduction in cell viability of 3T3-L1 preadipocytes are shown in Table 4. The untreated vehicle control samples were assumed 100% viable, while the positive control led to total cell lysis.

Generally, the results suggested some forms of dose-dependent alteration towards the viability of 3T3-L1 preadipocytes for all of the tested extract and fractions. The relatively low IC_20_ and IC_50_ values for RME, EAF and BUF in both MTT and NRU assays indicated potential cytotoxic, cytostatic or antiproliferative effects with greater concentration. The results, in conjunction with the report by Rayalam *et al.* [30], revealed the implication of phenolic compounds in inhibiting preadipocyte population growth and potential *in vivo* anti-obesity effects. A positive correlation between antioxidant activity of phenolic acids and its influence on cell population growth had also been mentioned [31]. Observations under the phase contract microscope revealed no indication of cellular toxicity with regard to morphologic appearance changes, at concentrations lower than 200 μg/mL for all of the tested extract and fractions. Taken together, a dosage concentration range up to 200 μg/mL was then used for the subsequent cellular antioxidant activity test.

### 2.6. Cellular Antioxidant Activity and Oxidative Stress

Free radicals are generated during the enzymatic reduction of oxygen for energy production by the mitochondria. They are, therefore, the byproducts of normal cellular metabolism. However, the production of intracellular ROS is closely associated with cellular response against oxidative stress. When ROS overwhelms the antioxidant defense system, oxidative stress occurs and leads to cellular damage. The hydroxyl radical and the peroxyl radical are among the major ROS generated in the cellular processes [1].

*In vivo* production for a majority of hydroxyl radicals occurs through catalytic breakdown of hydrogen peroxide, although it could occasionally be formed as a byproduct of immune action. Being highly reactive and having a very short half-life, the hydroxyl radical poses damage to almost all types of macromolecules and cannot be readily eliminated through enzymatic reaction. The peroxyl radical, on the other hand, is the most predominant free radical found in the human body. Being formed via the reaction of an oxygen and carbon-centered radical, the peroxyl radical is involved in an array of biological reactions, including DNA cleavage, lipid peroxidation and protein backbone modification [32].

**Figure 4:**
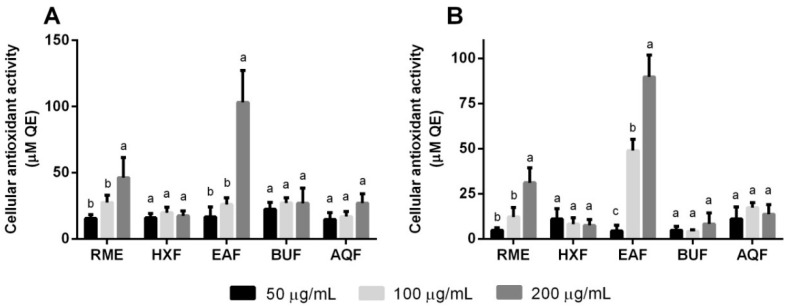
Cellular antioxidant activities under the influence of (**A**) 2,2′-azobis(2-amidinopropane) dihydrochloride (AAPH) and (**B**) H_2_O_2_. Data are expressed as the mean ± the standard deviation following triplicate experimentation. Different letters indicate significant differences (*p* < 0.05) within the different treatment concentrations. QE, quercetin equivalent.

Figure 4 depicts the effect of RME and its fractions on the cellular antioxidant activity (CAA) of 3T3-L1 preadipocytes under the stress influence of either H_2_O_2_ or 2,2′-azobis(2-amidinopropane) dihydrochloride (AAPH). The use of biologically-active hydroxyl and peroxyl radicals mimicked the damaging of cellular constituents in an actual biological system. In the present study, the localization of intracellular ROS was performed and presented as CAA. RME exhibited a protective effect on preadipocytes exposed to both peroxyl- and hydroxyl-induced oxidative stress at the concentration of 200 μg/mL, but not at lower concentrations. Overall, EAF appeared to possess the highest CAA against both radical-induced oxidative stress models. Additionally, the CAA exerted by EAF in the hydroxyl-induced oxidative stress happened in a dose-response manner, suggesting additive dosage action.

Adipocytes, being the main component of adipose tissue, govern homeostatic control over whole body metabolism. The formation of adipose tissue and fat mass is regulated by the differentiation of preadipocytes into mature adipocytes. Wang *et al.* [5] reported the involvement of mitochondrial ROS in the process of adipogenesis and its diminution with the presence of antioxidants. Another report by Marimoutou *et al.* [33] depicted the efficacy of polyphenols in controlling adipose tissue redox status and inflammation. Collectively, the elevated cellular antioxidant defense system by EAF indicated its potential ability to interfere with the adipogenesis process and inflammation. More explorations, nevertheless, are needed to support the findings.

Evaluating oxidative damage is often restricted by the chemically- or metabolically-unstable molecules that undergo constant turnover and repair. Intracellular 8-*iso* prostaglandin F2α (8-*iso*-PGF2α) levels have been demonstrated to possess more rapid recovery kinetics in comparison to its excretion [34]. Thus, the excretion of 8-*iso*-PGF2α in the supernatant of 3T3-L1 cells is being employed as an indicator for oxidative damage in the present study. The prostaglandin is produced through non-enzymatic peroxidation of the arachidonic acid precursor on the membrane phospholipids.

**Figure 5:**
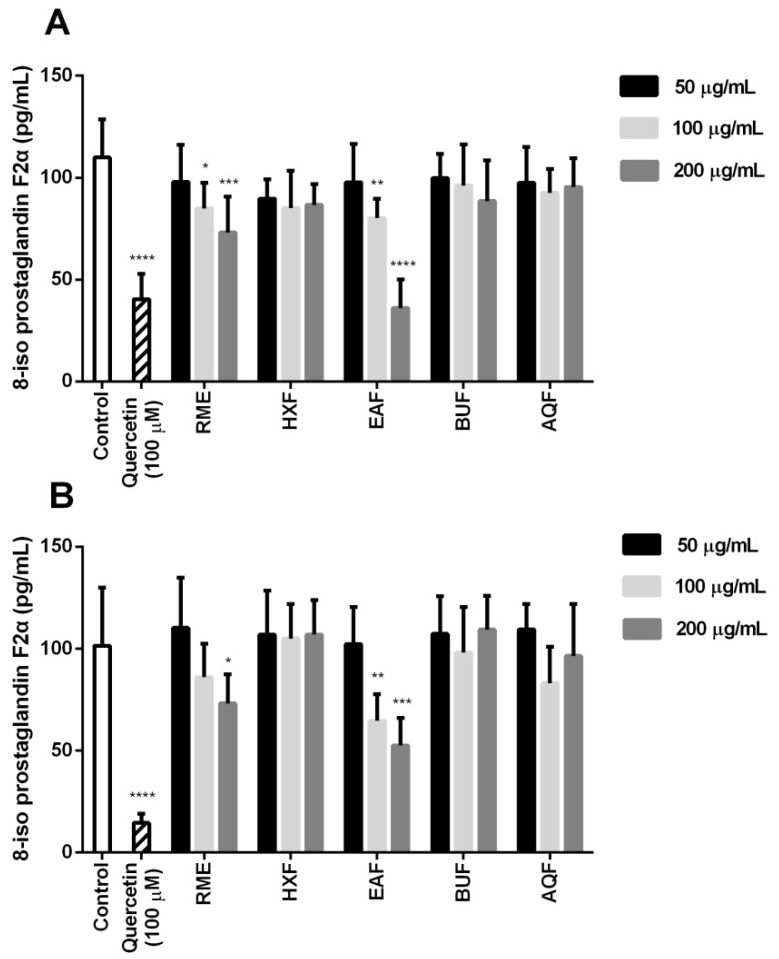
8-isoprostaglandin F2α concentrations under the influence of (**A**) AAPH and (**B**) H_2_O_2_. Data are expressed as the mean ± the standard deviation (*n* = 6) following triplicate experimentation. * *p* < 0.05, ** *p* < 0.01, *** *p* < 0.001 and **** *p* < 0.0001 compared to the control.

The levels of 8-*iso*-PGF2α under the effect of different extract and fractions are shown in Figure 5. Similarly, both RME and EAF demonstrated a dose-response ability to lower the concentration of the isoprostane. In particular, 200 μg/mL EAF equated the effect of 100 μM quercetin that acted as a positive control under the stress induced by peroxyl radicals. A correlational study also demonstrated significant correlations among TPC and TFC with CAA (0.94–0.98; *p* < 0.05) and isoprostane levels (0.90–0.93; *p* < 0.05). The data further reinstated the potential function of EAF in restoring the delicate balance between the reactive species and antioxidant defense through phenolic antioxidant enrichment.

### 2.7. Correlation between Phenolic Compounds and Antioxidant Activities

**Figure 6:**
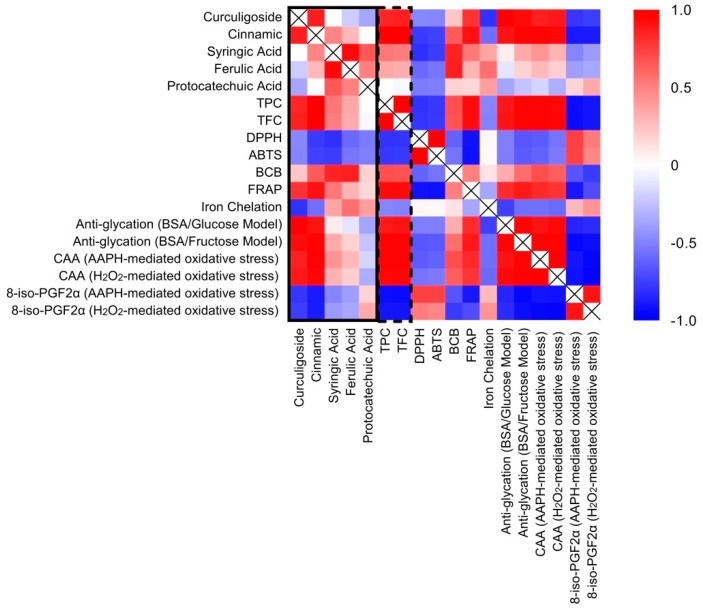
Correlation heat map of identified phenolic compounds and antioxidant activities. Each cell indicates the Pearson’s correlation coefficient value, *r*, for a pair of phenolic compound/s or antioxidant activities. Positive correlations (0 < *r* < 1.0) are displayed in red color, and negative correlations (−1.0 < *r* < 0) are displayed in blue color. Color intensity is proportional to the correlation coefficients.

In order to decipher and visualize the relationship between the identified phenolic compounds and antioxidant activities, a correlation heat map was produced using computed Pearson’s correlation coefficient values (Figure 6). Among the five phenolic compounds identified in the present study, protocatechuic acid demonstrated a relatively weak and moderate correlation with all of the tested antioxidant activities. The results contradicted previous findings that presented protocatechuic acid as an antioxidant having both hydrophilic and lipophilic capacities [35]. Zeraik *et al.* [36] reported that the esters of protocatechuic acid might act as a potent pro-oxidant that exacerbates redox cycling reactions. In the present case, the extraction and fractionation process with methyl alcohol and butyl alcohol might esterify protocatechuic acid, thereby provoking a profound influence on the pro-oxidant capacity. However, more experimental data are required to lend further support to this supposition.

Syringic acid and ferulic acid depicted a similar weak correlation trend. Nevertheless, both compounds correlated well with BCB activity (0.84–0.86; *p* = 0.07), even though it was not statistically significant. Additionally, syringic acid also displayed moderately strong correlations with the ability to scavenge the DPPH radical (0.80; *p* = 0.10) and the ABTS radical cation (0.76; *p* = 0.14). The results were in accordance with the report by Natella *et al.* [37] that stated the structure-activity relation of phenolic compounds. In particular, the aromatic substitution influenced the antioxidative activity of these compounds. Being a benzoic acid derivative bearing *p*-hydroxydimethoxy substitution, syringic acid had an antioxidant capacity higher than that of ferulic acid, a *p*-hydroxymethoxy acid derived from cinnamic acid. The antioxidant capacities of both compounds were also higher when compared to protocatechuic acid, a benzoic acid-derived dihydroxy acid.

Significant correlations between curculigoside and anti-glycation activities (0.92–0.99; *p* < 0.05), as well as CAA under the influence of H_2_O_2_ (0.89; *p* < 0.05) were also identified. While curculigoside might not possess efficient chemical reactivity towards free-radical quenching and reducing, the compound might exert protection against oxidative damage in the biological system. Curculigoside has been reported to exert a protective effect against intracellular ROS production in primary mouse cortical neuronal culture, primary rat osteoblast and human umbilical vein endothelial cells [38,39,40]. Its antioxidant effect has potentially afforded enhanced learning performance and amelioration of bone loss in mutated transgenic mice of Alzheimer’s disease [41].

Comparatively, cinnamic acid showed the strongest correlations with all of the tested antioxidant activities. Specifically, the compound correlated significantly with the FRAP assay (0.907; *p* < 0.05), anti-glycation activities (0.90–0.96; *p* < 0.05), CAA (0.94–0.98; *p* < 0.05), as well as isoprostane levels (0.88–0.89; *p* < 0.05). The present data were supported by previous findings that reported moderate radical quenching activities of cinnamic acid owing to the absence of the hydroxyl group on the aromatic ring [42,43]. The ability of cinnamic acid to undergo a reduction mechanism, nevertheless, afforded the strong positive correlations between the compound with the FRAP assay and anti-glycation activities [44]. Moreover, the protective effects of cinnamic acid in modulating ROS generation or degradation coincided with previous experimental reports employing both *in vitro* and *in vivo* models [45,46].

In general, individual phenolics differed in their contribution to the antioxidant properties, potentially due to varying concentrations and the structure-antioxidant activity relationships. The TPC and TFC, however, displayed significant strong correlations with most antioxidant activities tested in the present study. Wang *et al.* [47] suggested that differing combinations of bioactive ingredients would alter the total antioxidant efficacies through synergistic, complementary or antagonistic interactions. In the present case, the better efficacy of EAF compared to RME might relate to the synergistic/complementary effect among the enriched phenolics content, in particular curculigoside and cinnamic acid.

## 3. Materials and Methods

### 3.1. Chemicals

Methanol, *n*-hexane, *n*-butanol, ethyl acetate, chloroform, Triton X-100 and Tween 20 were purchased from Fisher Scientific (Loughborough, Leicestershire, UK). Linoleic acid, gallic acid, quercetin, β-carotene (Type I synthetic, 95%), bovine serum albumin (BSA), ABTS, potassium persulfate, ferrous sulfate, hydrogen peroxide, AAPH, tetramethylchroman-2-carboxylic acid (trolox), ethylenediamine tetra acetic acid (EDTA), DPPH, dichloro-dihydro-fluorescein diacetate (DCFH-DA), Folin–Ciocalteu’s phenol reagent and ferrozine were supplied by Sigma-Aldrich Co. (St. Louis, MO, USA). Dulbecco’s Modified Eagle’s Medium (DMEM), gentamicin and fetal bovine serum (FBS) were purchased from GIBCO (Grand Island, NY, USA). The 8-iso prostaglandin F2α ELISA kit was bought from Cusabio (Wuhan, China). MTT and neutral red were obtained from Nacalai Tesque (Kyoto, Japan). All of the other chemicals were purchased from Sigma-Aldrich Co.

### 3.2. Procurement and Preparation of Plant Materials

*M. latifolia* rhizomes were collected from Beranang, Selangor, Malaysia (Geographical Coordinates: 2.8833° N, 101.8667° E). A voucher specimen of the plant (SK 1709/09) was confirmed and deposited in the Biodiversity Unit, Institute of Bioscience, Universiti Putra Malaysia, Serdang, Malaysia. The rhizomes were oven dried at 40 °C, pulverized and passed through a 30-mesh sieve. The sieved materials were subsequently stored at −20 °C till further analysis.

### 3.3. Extraction and Fractionation

The extraction and fractionation of *M. latifolia* rhizome were performed in triplicate. One hundred grams of *M. latifolia* rhizome powder were extracted with 1 L of 80% methanol. The extraction process was performed for 1 h in a sonication bath followed by filtration through Whatman filter paper No. 1. The procedure was repeated twice on the residues. Eventually, the RME was obtained after reduced pressure solvent removal (Rotavapor R210, Buchi, Postfach, Flawil, Switzerland) and lyophilization (Virtis Benchtop K Freeze Dryer, SP Industries, Warminster, PA, USA).

Bio-guided fractionation was performed on RME by solubilizing 5 g of RME in water and sequentially partitioned with an equal volume of hexane, ethyl acetate and *n*-butanol to yield their respective fractions, *i.e.*, HXF, EAF and BUF. The partition process for each solvent was repeated thrice before further fractionation with the next solvent. The residue of the sequential partition process served as the AQF. Finally, solvents from all obtained fractions were removed via evaporation or lyophilization. The yields of RME, HXF, EAF, BUF and AQF were measured prior to storage at −20 °C till further analyses.

### 3.4. Determination of Total Phenolic and Total Flavonoid Contents

Total phenolic content (TPC) of the *M. latifolia* rhizome extract and fractions was determined by the Folin–Ciocalteu reagent assay described by Ooi *et al.* [48]. Gallic acid was used as a positive control, and the TPC of the samples was expressed as milligrams gallic acid equivalents (mg GAE)/g extract or fraction. Total flavonoid content (TFC) of the extract and fractions was evaluated adopting the aluminum chloride colorimetric assay with a modified experimental procedure suitable for use with 96-well plates [48]. Rutin was used as positive control, and the TFC of the samples was expressed as milligrams rutin equivalents (mg RE)/g extract or fraction. 

### 3.5. HPLC-DAD Analysis for Phenolic Compounds

Identification and quantification of major phenolic compounds in the RME, HXF, EAF, BUF and AQF were conducted by performing HPLC-DAD analysis following a modified method proposed by Zhang *et al.* [49]. The chromatographic separations were conducted on Agilent Zorbax SB C-18 column (5 μm, 150 mm × 4.6 mm) using an Agilent 1200 series HPLC (Agilent, Stevens Creek Blvd, Santa Clara, CA, USA) system equipped with an auto-sampler and diode array detector. The column was kept at 30 °C with a flow rate of 0.8 mL/min during the analysis. All of the solutions were filtered through a 0.22-μm Millipore syringe filter, type GV (Millipore, Bedford, MA, USA), and the mobile phase was degassed prior to HPLC injection. The sample injection volume was 20 μL.

The solvent system employed was a gradient of water–acetic acid (97:3, *v*/*v*) (A) and acetonitrile (B) as: 0.0%–8.5% B for 5 min; 8.5%–2.0% B for 11.5 min; 2.0%–18.0% B for 18.5 min; 18%–20% B for 15 min; 20%–30% B for 15 min; and finally, 30.0%–0.0% for another 5 min. The absorbance of the specific wavelengths (280 and 320 nm) was acquired, and the chromatograms were integrated by the Agilent Chemstation enhanced integrator. The identification and quantification of phenolic compounds were achieved by spiking, comparing retention time and plotting the standard curve using commercially available phenolic compounds (Sigma-Aldrich Co.). Calibration standards of phenolic compounds were prepared in the range of 1.95–250.00 μg/mL using eight concentration levels. The analyses for all samples were performed in triplicate, and the results were expressed as milligrams per gram (mg/g) of extract or fraction.

### 3.6. Antioxidant Activity Assays

#### 3.6.1. DPPH Radical Scavenging Activity

The DPPH radical scavenging activity of the extract and fractions was determined following the procedure described by Chan *et al.* [50] with some modifications. The absorbance of each sample was recorded at 540 nm spectrophotometrically. The percentage inhibition of the samples was calculated as:

% Inhibition = (1 − A_S_/A_0_) × 100 (1)

where A_0_ is the absorbance of blank and A_S_ is the absorbance of the sample mixture. Trolox was used as a positive reference, and IC_50_ values denote the concentration of the sample required to scavenge 50% of the DPPH radical.

#### 3.6.2. ABTS Radical Cation Scavenging Activity

The ABTS radical cation scavenging activity was measured according to the methods by Chan *et al.* [50]. The spectrophotometric absorbance of the sample mixture was recorded at 734 nm after 10 min. The percentage inhibition of the samples was calculated as:

% Inhibition = (1 − A_S_/A_0_) × 100 (2)

where A_0_ is the absorbance of the blank and A_S_ is the absorbance of the sample mixture. Trolox was used as a positive reference, and IC_50_ values denote the concentration of the sample required to scavenge 50% of the ABTS radical cation.

#### 3.6.3. BCB Assay

Antioxidant activity of the extract and fractions was assessed by the modified BCB assay as reported previously [50]. The initial and final absorbance of reaction mixtures was measured at 470 nm spectrophotometrically. The percentage inhibition of the samples was calculated as:

% Inhibition = [(A_S(t=120)_ − A_C(t=120)_)/(A_C(t=0)_ − A_C(t=120)_)] × 100 (3)

where A_S(t=120)_ is the absorbance of the sample at 120 min, A_C(t=120)_ is the absorbance of the control at 120 min and A_C(t=0)_ is the absorbance of control at 0 min. Trolox was used as a positive reference, and the result was expressed as μg trolox equivalent (TRE)/mg extract.

#### 3.6.4. FRAP Assay

The FRAP value of the tested samples was determined using a modified ferricyanide method. Briefly, 1 mL of properly-diluted samples was sequentially added with 5 mL of deionized distilled water, 1.5 mL of 1 M HCL, 1.5 mL of potassium ferricyanide (1% *w*/*v*), 0.5 mL sodium dodecyl sulfate solution (1% *w*/*v*) and 0.5 mL of ferric chloride (0.2% *w*/*v*). Following incubation at 50 °C for 20 min, the absorbance of the cooled 10-mL reaction mixtures was read at 750 nm against the blank. Trolox was used as the standard, and FRAP was expressed as μg trolox equivalent (TRE)/mg extract.

#### 3.6.5. Iron Chelating Activity

The ability of the extract and fractions to chelate ferrous ions was determined according to the method described by Yusri *et al.* [51]. Na_2_EDTA was used as a positive reference, and IC_50_ values denote the 50% inhibitory concentration of the sample for ferrozine-Fe^2+^ complex formation.

### 3.7. Non-Enzymatic Anti-Glycation Activities

The anti-glycation efficacy of the extract and fractions was evaluated using the modified BSA/glucose and BSA/fructose model systems described by Mahomoodally *et al.* [52]. The fluorescent intensity was quantified using a fluorescence microplate reader (Biotek Synergy H1 Multi-Mode Reader, Winooski, VT, USA) with an excitation/emission wavelength pair of 370/440 nm. The percentage inhibition of glycation was calculated as:

% Inhibition = [1 − (FLS – FLB)/(FLC – FLB)] × 100
(4)

where FLS is the fluorescence intensity of the sample mixture, FLB is the fluorescence intensity of the blank mixture and FLC is the fluorescence intensity of the control mixture. Catechin was employed as a positive reference, and the result was expressed as μM catechin equivalent (CE).

### 3.8. Cell Culture

3T3-L1 cells, a type of mouse embryonal fibroblasts, were obtained from American Type Culture Collection. The cells were maintained in Dulbecco’s Modified Eagle Medium (DMEM) supplemented with 10% fetal bovine serum and 100 μg/mL gentamicin at 37 °C in a humidified, 5% CO_2_ atmosphere. The passage number range for the cell line was maintained between 20 and 25.

#### 3.8.1. Cell Viability Assays

The cell viability studies were conducted using both MTT and neutral red colorimetric assays. The MTT assay was carried out according to the method described previously by Foo *et al.* [53], with minor modifications. At the end of a 24-h exposure period with test samples, cells were further incubated for 4 h with 20 μL of MTT (5 mg/mL). After PBS rinse, 200 μL of DMSO were added to solubilize the formazan crystal. Absorbance measurements were made at 570 nm spectrophotometrically.

The NRU assay was performed based on the modified protocol described by Kamba *et al.* [54]. After 24 h of exposure to the test samples, the cells were incubated with 100 μg/mL neutral red dye dissolved in serum-free medium for 2 h at 37 °C. Following the rinse with phosphate buffer saline (PBS), the cells were added with elution medium consisting of 50% ethanol and 1% acetic acid. The cells were then agitated gently for 10 min to achieve complete dissolution, and lysosomal uptake of neutral red was measured spectrophotometrically at 540 nm.

DMSO (0.1%; *v*/*v*) and Triton X-100 (1.0%; *v*/*v*) in DMEM were respectively being employed as untreated vehicle control and positive control. The viabilities of cells for both MTT and neutral red assays were expressed as the fraction of viable cells relative to untreated vehicle control cultures. The IC_20_ and IC_50_ values (μg/mL), indicating the respective concentration of test sample producing 20% and 50% cell viability, were calculated using GraphPad Prism 6.01 (San Diego, CA, USA) with the 4 parameter logistic function standard curve analysis.

#### 3.8.2. Cellular Antioxidant Activity

The production of intracellular ROS was measured by the DCFH-DA assay following the method reported by Ramful *et al.* [55] with slight modifications. 3T3-L1 cells, at the confluent state, were first incubated with properly-diluted test samples for 24 h in DMEM containing 0.5% FBS. After being rinsed twice with PBS, the cells were incubated with 10 μM DCFH-DA in PBS for 30 min at 37 °C in 5% CO_2_. All of the wells were then filled with 100 μL PBS following another round of wash with PBS to rinse away the free DCFH-DA. Subsequently, 1 mM AAPH or 4 mM H_2_O_2_ was added to act as the source of free radicals. The fluorescent intensity was immediately quantified using a fluorescence microplate reader with an excitation wavelength of 485 nm and an emission wavelength of 530 nm. Quantification of fluorescent intensity was monitored for up to 2 h with 5-min intervals in between. The integrated area under the curve (AUC) and cellular antioxidant activity (CAA) values for each sample and standard were calculated as:

AUC = 0.5 + (RFU_1_/RFU_0_) + (RFU_2_/RFU_0_) + (RFU_3_/RFU_0_) + …… + 0.5(RFU_n_/RFU_0_) (5)

CAA units = 100 − (AUC_Sample_/AUC_Control_) × 100 (6)

where RFU_0_ is the relative fluorescent value at time zero, RFU_n_ is the final relative fluorescent value, AUC_Sample_ is the integrated area under the curve for cells incubated with samples or standards and AUC_Control_ is the integrated area under the curve for cells without sample treatment. Quercetin was employed as a positive reference, and the result was expressed as μM quercetin equivalent (QE).

#### 3.8.3. 8-*iso*-PGF2α Content

Confluent 3T3-L1 cells were first pre-incubated for 24 h in DMEM containing 0.5% FBS with different dilutions of test samples. After the pre-incubation, cells were then treated with either 200 μM AAPH or 200 μM H_2_O_2_ for 4 h. The cell culture media were then collected, and quantification of 8-*iso*-PGF2α was performed using an enzyme immunoassay kit (Cusabio, China) according to the manufacturer’s instructions. Quercetin was employed as positive reference, and the 8-*iso*-PGF2α levels were reported as pg/mL of media.

### 3.9. Statistical Analysis

Data are reported as the mean ± the standard deviation from triplicate experimentations. Analysis of variance (ANOVA) accompanied with Tukey’s multiple comparison tests (GraphPad Prism 6.01) were performed, and significance was established at *p* ≤ 0.05. Pearson’s correlation coefficient test was conducted to identify pairwise correlations between phenolic compounds and the antioxidant activities of tested samples. The correlation heat map was generated by GraphPad Prism 7.00 using the computed Pearson’s correlation coefficient values.

## 4. Conclusions

The present findings showed that the contributions of individual phenolic compounds to the antioxidant activities were predominately concentration- and structure-dependent. The enriched phenolic content, particularly curculigoside and cinnamic acid, might account for the enhanced antioxidant attributes and protective effect against oxidative damage afforded by EAF (curculigoside-cinnamic acid-rich fraction). In order to better understand the synergistic/complementary effect of the phenolic constituents within the fraction, individual and compounding studies of these chemical constituents are recommended. *In vivo* evidence-based studies, safety, bioavailability and standardization of the curculigoside-cinnamic acid fraction might further warrant its potential use as antioxidant therapeutic agents.

## Figures and Tables

**Table 1:** Extract/fraction yield, total phenolic content and total flavonoid content.

Extract or Fraction	Yield (g/100 g Dry Weight)	Total Phenolic Content (mg GAE/g Extract or Fraction)	Total Flavonoid Content (mg RE/g Extract or Fraction)
RME	7.95 ± 0.02 ^d^	48.35 ± 10.54 ^b^	21.97 ± 3.33 ^b^
HXF	0.71 ± 0.02 ^e^	0.30 ± 0.27 ^e^	0.18 ± 0.10 ^d^
EAF	10.38 ± 0.13 ^c^	187.30 ± 9.28 ^a^	76.77 ± 2.68 ^a^
BUF	36.37 ± 0.29 ^b^	24.96 ± 4.34 ^c^	10.84 ± 2.05 ^c^
AQF	42.54 ± 0.59 ^a^	4.62 ± 0.85 ^d^	1.62 ± 0.63 ^d^

Data are expressed as the mean ± the standard deviation (*n* = 6); different letters within the same column indicate significant differences (*p* < 0.05); GAE, gallic acid equivalent; RE, rutin equivalent.

**Table 2:** Phenolic compositions by HPLC-DAD analysis.

Phenolic Compound	Retention Time (min)	Individual Phenolic Content (mg/g Extract or Fraction)				
		RME	HXF	EAF	BUF	AQF
Gallic acid	3.835 ± 0.014	Nd	Nd	Nd	Nd	Nd
Protocatechuic acid	6.391 ± 0.019	1.75 ± 0.27 ^b^	Nd	Nd	4.58 ± 0.09 ^a^	0.11 ± 0.02 ^c^
*p*-Hydroxybenzoic acid	9.135 ± 0.012	Nd	Nd	Nd	Nd	Nd
Gentisic acid	9.962 ± 0.011	Nd	Nd	Nd	Nd	Nd
Chlorogenic acid	11.917 ± 0.013	Nd	Nd	Nd	Nd	Nd
Vanillic acid	14.211 ± 0.012	Nd	Nd	Nd	Nd	Nd
Caffeic acid	18.158 ± 0.014	Nd	Nd	Nd	Nd	Nd
Syringic acid	25.075 ± 0.018	4.69 ± 0.09 ^a^	Nd	1.75 ± 0.02 ^c^	3.26 ± 0.03 ^b^	Nd
*p*-Coumaric acid	36.165 ± 0.013	Nd	Nd	Nd	Nd	Nd
Ferulic acid	38.534 ± 0.019	2.47 ± 0.04 ^a^	0.09 ± 0.03 ^d^	0.29 ± 0.06 ^c^	1.06 ± 0.04 ^b^	Nd
Sinapic acid	43.609 ± 0.013	Nd	Nd	Nd	Nd	Nd
Salicylic acid	48.985 ± 0.015	Nd	Nd	Nd	Nd	Nd
Curculigoside	51.353 ± 0.021	6.47 ± 0.04 ^b^	Nd	90.49 ± 0.04 ^a^	Nd	Nd
Cinnamic acid	59.323 ± 0.020	7.05 ± 0.01 ^b^	Nd	12.43 ± 0.10 ^a^	4.39 ± 0.06 ^c^	1.70 ± 0.08 ^d^
Sum of Phenolic Content		22.43 ^b^	0.09 ^d^	104.96 ^a^	13.29 ^b^	1.81 ^c^

Data are expressed as the mean ± the standard deviation (*n* = 3); different letters within the same row indicate significant differences (*p* < 0.05); Nd, not detected.

**Table 3:** Antioxidant activities of *M. latifolia* rhizome methanolic extract and its fractions.

Extract or Fraction	DPPH Radical Scavenging Activity	ABTS Radical Cation Scavenging Activity	β-Carotene Bleaching Activity	Ferric Reducing Antioxidant Power	Iron Chelating Activity
	IC_50_ (μg/mL)	IC_50_ (μg/mL)	(μg TRE/mg Extract or Fraction)	(μg TRE/mg Extract or Fraction)	IC_50_ (mg/mL)
RME	90.76 ± 2.48 ^c^	431.00 ± 36.90 ^c^	3.97 ± 0.22 ^a^	204.44 ± 16.66 ^b^	6.13 ± 1.35 ^a^
HXF	495.47 ± 24.04 ^b^	1588.50 ± 261.61 ^b^	0.57 ± 0.04 ^e^	126.90 ± 13.54 ^c^	5.72 ± 1.88 ^a^
EAF	36.96 ± 1.10 ^c^	132.24 ± 19.20 ^c,d^	2.34 ± 0.06 ^b^	308.94 ± 16.98 ^a^	1.90 ± 0.04 ^c^
BUF	93.36 ± 0.38 ^c^	232.60 ± 14.68 ^c,d^	1.61 ± 0.09 ^c^	198.36 ± 18.46 ^b^	5.01 ± 2.29 ^a,b^
AQF	707.41 ± 143.29 ^a^	2537.98 ± 379.22 ^a^	1.34 ± 0.17 ^d^	57.68 ± 6.64 ^d^	3.55 ± 0.02 ^b,c^
Trolox	23.87 ± 0.40 ^c^	20.54 ± 0.07 ^d^			
Na_2_EDTA					0.03 ± 0.00 ^d^

Data are expressed as the mean ± the standard deviation (*n* = 6); different letters within the same column indicate significant differences (*p* < 0.05); TRE: trolox equivalent.

**Table 4:** IC_20_ and IC_50_ values obtained by the MTT and NRU assays.

	MTT		NRU	
	IC_20_ (μg/mL)	IC_50_ (μg/mL)	IC_20_ (μg/mL)	IC_50_ (μg/mL)
RME	155.93 ± 9.83 ^d^	561.28 ± 69.53 ^c^	206.85 ± 15.57 ^c^	663.43 ± 88.62 ^b^
HXF	448.49 ± 21.26 ^a^	>2000 ^a^	522.59 ± 27.74 ^a^	>2000 ^a^
EAF	111.73 ± 9.57 ^e^	509.59 ± 49.75 ^c^	148.11 ± 16.23 ^d^	536.99 ± 45.94 ^c^
BUF	192.65 ± 12.33 ^c^	649.97 ± 53.82 ^b^	229.63 ± 20.12 ^c^	729.65 ± 61.27 ^b^
AQF	413.73 ± 28.75 ^b^	>2000 ^a^	382.59 ± 27.66 ^b^	>2000 ^a^

Data are expressed as the mean ± standard deviation (*n* = 6); different letters within the same column indicate significant differences (*p* < 0.05).

## Abbreviations

The following abbreviations are used in this manuscript:

RME	rhizome methanolic extract
HXF	hexane fraction
EAF	ethyl acetate fraction
BUF	*n*-butanol fraction
AQF	aqueous fraction
GAE	gallic acid equivalent
RE	rutin equivalent
CE	catechin equivalent
TRE	trolox equivalent
NRU	neutral red uptake
CAA	cellular antioxidant activity
AUC	area under the curve
